# Differences in the Curing of [*PSI^+^*] Prion by Various Methods of Hsp104 Inactivation

**DOI:** 10.1371/journal.pone.0037692

**Published:** 2012-06-18

**Authors:** Yang-Nim Park, David Morales, Emily H. Rubinson, Daniel Masison, Evan Eisenberg, Lois E. Greene

**Affiliations:** 1 Laboratory of Cell Biology, National Heart Lung Blood Institute, National Institutes of Health, Bethesda, Maryland, United States of America; 2 Laboratory of Biochemistry and Genetics, National Institute of Diabetes, Digestive, and Kidney Diseases, National Institute of Health, Bethesda, Maryland, United States of America; University of Kent, United Kingdom

## Abstract

[*PSI* ^+^] yeast, containing the misfolded amyloid conformation of Sup35 prion, is cured by inactivation of Hsp104. There has been controversy as to whether inactivation of Hsp104 by guanidine treatment or by overexpression of the dominant negative Hsp104 mutant, Hsp104-2KT, cures [*PSI* ^+^] by the same mechanism– inhibition of the severing of the prion seeds. Using live cell imaging of Sup35-GFP, overexpression of Hsp104-2KT caused the foci to increase in size, then decrease in number, and finally disappear when the cells were cured, similar to that observed in cells cured by depletion of Hsp104. In contrast, guanidine initially caused an increase in foci size but then the foci disappeared before the cells were cured. By starving the yeast to make the foci visible in cells grown with guanidine, the number of cells with foci was found to correlate exactly with the number of [*PSI^+^*] cells, regardless of the curing method. Therefore, the fluorescent foci are the prion seeds required for maintenance of [*PSI^+^*] and inactivation of Hsp104 cures [*PSI^+^*] by preventing severing of the prion seeds. During curing with guanidine, the reduction in seed size is an Hsp104-dependent effect that cannot be explained by limited severing of the seeds. Instead, in the presence of guanidine, Hsp104 retains an activity that trims or reduces the size of the prion seeds by releasing Sup35 molecules that are unable to form new prion seeds. This Hsp104 activity may also occur in propagating yeast.

## Introduction

Prions are infectious proteins that self-propagate by changing from their normal folded form to a misfolded amyloid form. In mammals, prions are implicated in various fatal neurodegenerative diseases, such as scrapie in sheep, bovine spongiform encephalopathy in cattle, and Crueutzfeld-Jacob disease in humans. Prions have also been found in the yeast *Saccharomyces cerevisiae* and in the fungus *Podospora anserina.* Yeast prions have served as convenient models to study the mechanism of prion propagation. The misfolded yeast prion is transmitted as seeds from mother to daughter during cell division, and these seeds then cause conversion of the properly folded protein to the misfolded prion form [1].

One of the best characterized yeast prions, [*PSI* ^+^], occurs when the translation termination factor Sup35 is transformed into its aggregated amyloid form. Sup35 has three domains, N, M, and C, which are respectively an N-terminal Asn/Gln rich aggregation-prone domain, a middle domain, and a C-terminal domain that mediates translation termination. The [*PSI* ^+^] phenotype has impaired termination of protein translation as opposed to the [*psi* ^-^] phenotype in which Sup35 is present in its properly folded form. Maintenance of [*PSI* ^+^] and other misfolded yeast prions requires molecular chaperones, in particular Hsp104 [2], [3]. Hsp104, a member of the triple-A ATPase family, is a hexameric protein with two nucleotide binding domains (NBD1 and NBD2) in each of its 6 molecular subunits [4]. Inactivation of its ATPase activity, either by incubating yeast with 5 mM guanidine or by overexpressing the dominant negative Hsp104 mutant (Hsp104-2KT), with mutations in both of its NBD domains (K218T and K620T), cures the prion phenotype [2], [5], [6], [7].

One of the techniques that have been employed to understand the role of Hsp104 in prion propagation is confocal microscopy. Initially, the truncated Sup35 fluorescent fragment, NMG, was used as a reporter of the [*PSI* ^+^] phenotype [7], [8], [9], [10], [11]. NMG is diffusive in [*psi^-^*] yeast, whereas it forms fluorescent foci in [*PSI* ^+^] yeast. However, NMG does not retain the functional properties of the parent molecule. More recently, Sup35 has been engineered with a GFP inserted between its N- and M- domains to produce NGMC [12], [13], which still retains both the functional and conformational integrity of the parent molecule. Similar to NMG, NGMC appears diffusive in [*psi^-^*] yeast and forms foci in [*PSI* ^+^] yeast. Consistent with this difference in appearance, the diffusional mobility of NGMC is much faster in [*psi^-^*] than in [*PSI* ^+^] yeast [12].

To understand the role of Hsp104 in prion propagation, changes in both NMG and NGMC fluorescence have been examined following inactivation of Hsp104 in [*PSI* ^+^] yeast. The Chernoff laboratory observed that when NMG was expressed from the *SUP35* promoter on a centromeric plasmid, large fluorescent NMG foci were observed when [*PSI* ^+^] was cured by overexpressing Hsp104-2KT [7], [14]. In contrast, during curing of [*PSI* ^+^] by guanidine treatment, they observed that the NMG fluorescence became diffusive [7]. Furthermore, curing of [*PSI* ^+^] by overexpression of Hsp104-2KT was much faster than curing by guanidine treatment [7], [14]. Based on the differences in curing of kinetics and NMG fluorescence, they concluded that guanidine treatment cures [*PSI* ^+^] by a different mechanism than overexpression of Hsp104-2KT. In contrast, Tuite and coworkers found that inactivation of Hsp104 by guanidine treatment or overexpression of Hsp104-2KT cured [*PSI* ^+^] with similar kinetics [15], [16]. On this basis, they proposed a model in which inactivation of Hsp104 either by guanidine treatment or by overexpression of Hsp104-2KT inhibits severing, which in turn prevents generation of new seeds. Curing of prion then occurs when the seeds are diluted out by cell division [15], [16], [17], [18].

Two recent microscopy studies have shown a defect in the transmission of prion seeds from mother to daughter during the curing of [*PSI* ^+^] by guanidine treatment. This defect in transmission was observed either when NGMC was expressed from the *MFA1* promoter [19] or when NMG was expressed from the *GAL1* promoter [20]. In contrast, results from our laboratory using NGMC expressed from the *SUP35* promoter showed that during curing by guanidine treatment, the NGMC foci became diffusive, acquiring mobility similar to their mobility in [*psi^-^*] yeast even though the yeast were still [*PSI* ^+^] [21].

In the present study, we reinvestigated the changes in NGMC fluorescence that occur when [*PSI^+^*] yeast were cured either by overexpression of Hsp104-2KT or by guanidine treatment to better understand the role of Hsp104 in prion propagation. Our data showed that even though the seeds appeared to become much smaller during curing by guanidine treatment than by overexpression of Hsp104-2KT, the number of cells with foci correlated exactly with the number of [*PSI* ^+^] cells regardless of the method of curing. These results showed that the NGMC fluorescent foci are indeed the prion seeds that maintain [*PSI^+^*] and curing occurs because of inhibition of the severing activity of Hsp104. In addition, when [*PSI^+^*] was cured by depleting the yeast of Hsp104, the NGMC foci remained prominent, which establishes that the loss of detectable foci during guanidine treatment is due to an Hsp104-dependent activity. Measurements of the seed size showed that the loss of detectable seeds occurred because the seeds became much smaller during the curing of [*PSI* ^+^] by guanidine treatment than by overexpression of Hsp104-2KT. Therefore, our data are consistent with the thesis that during curing by guanidine treatment, Hsp104 retains an activity that reduces the size of the seed without changing their number of seeds.

## Materials and Methods

### Yeast Strains, Plasmid, Media, and Growth Conditions

The 1074 [*PSI^+^*] yeast strain, used routinely throughout this study, has *SUP35-GFP* (*NGMC*) integrated into the genomic locus of *SUP35*. This strain was derived from wild-type strain 779-6A (*MAT* **a**, *kar1*-*1*, *SUQ5*, *ade2*-*1*, *his3*Δ*202*, *leu2*Δ*1*, *trp1*Δ*63*, *ura3*-*52*) [6]. The L2885 yeast strain (*MAT* **α**, *kar1*-*1*, *SUQ5*, *ade2*-*1*, *his3*Δ*202*. *trp1*Δ*63*, *ura3*-*52*, *leu2*-*3*, 112, *sup35*::*SUP35*-*GFP*) is a weak [*PSI* ^+^] [*pin^-^*] strain derived from 74-D694 strain (strain from S. Liebman, Univ. of Illinois) [22]. Plasmid pFL39-GAL-HSP1042KT [7], which is a *TRP1*-based centromeric plasmid and has *HSP104* containing two amino acid mutations (K218T and K620T) under the control of *GAL1* promoter, was transformed into these strains as described [23]. Single point mutants of Hsp104, Hsp104(K218T) and Hsp104(K620T), were expressed under the control of *GAL1* promoter in yeast transformed with either pRS314-GAL1-HSP104(K218T) or pRS314-GAL1-HSP104(K620T). Plasmid pRS314-GAL1-SSA1 contains the *SSA1* coding region with its terminator region (287 bp) fused to *GAL1* promoter. *GFP* on a centromeric plasmid pJ543 was expressed by the *SUP35* promoter in the 779-6A strain.

The *HSP104* gene was conditionally deleted by using the FLP-FRT recombination system [24]. PCR amplified *Bam*HI-*Xho*I *HSP104* fragment was cloned in pRS315 to generate pRS315-FRT-HSP104. The plasmid pRS314-GAL1-FLP was generated by cloning of *Xho*I-*Sac*I fragment from the plasmid pC5GAL1-FLP into pRS314. All sequences have been verified. Under non-inducing condition of Flp recombinase, the yeast *hsp10*4 deletion (Δ*hsp10*4) strain derived from 1074 with plasmids pRS315-FRT-HSP104 and pRS314-GAL1-FLP was able to maintain [*PSI* ^+^] as well as the Δ*hsp10*4 strain with the wild type copy of *HSP104* on centromeric plasmid pJ312 [25]. The Hsp104 protein level in the latter strains was the same as the endogenous level.

Yeasts were grown at 30°C on synthetic defined medium (SD; 0.7% yeast nitrogen base, 2% glucose) with complete supplement mixture (CSM) or the appropriate amino acid dropout complete supplement mixture for selection and maintenance of the particular plasmid. Synthetic galactose (SGal) medium contains both 2% galactose and 2% raffinose in place of glucose. ½YPD solid medium used in the plating assays contains 0.5% yeast extract, 2% peptone, and 2% glucose. Yeast were grown in synthetic medium rather than yeast peptone medium since the latter medium had background fluorescence that interfered with imaging NGMC. Cultures were always maintained in active growing conditions (OD_600_≤0.6) by periodic dilution with fresh medium.

### Curing Experiments

To express the different mutants of Hsp104, cells from growing culture in SD medium were shifted to SGal medium and continued to grow until curing of [*PSI* ^+^] prion. Cells were grown in the presence of 5 mM guanidine hydrochloride (Sigma, St. Louis) to cure [*PSI* ^+^] either in SD or SGal, as indicated. Yeast were grown in SGal to induce expression of the different Hsp104 mutants and Flp recombinase. To determine the extent of curing, yeast were plated on ½YPD plates as described previously [21]. Colonies with any white sectors were counted as [*PSI* ^+^].

### Confocal Microscopy and Foci Counting

Cells were imaged on a Zeiss live confocal microscope using a piezoelectric stage to obtain Z-stacks with a 100X objective in 8-well 25-mm^2^ chambered coverslips (Lab-Tek, Rochester). The same confocal settings were used for all images. To count cells with foci and number of foci in a cell, cells at indicated generations were fixed in 4% paraformaldehyde (PFA) (Sigma, St. Louis) in phosphate buffer saline for 5 min at room temperature, followed by acquiring Z-stack images (16 slices at 0.4 µm intervals). In order to count the foci in cells grown with 5 mM guanidine, cells were either incubated for 1 h in water or for 1–1.5 h in SGal medium to induce expression of Hsp104-2KT. To image bud scars, cells were stained with 2 µg/ml Alexa Fluor 647 conjugated wheat germ agglutinin (WGA) from Invitrogen for 20 min at room temperature. The transmission of the prion NGMC foci from mother into daughter cells was imaged using a Zeiss confocal microscope LSM780. Daughter cells were photo-bleached for 5 s before monitoring. Imaging was always performed on log phase yeast cells (OD_600_≤0.6) to prevent the development of fluorescent vacuoles, which can be confused with fluorescent foci.

### Individual Cell Imaging and Plating

To determine the prion phenotype of the progeny originating from individual cells, a 0.5 µl drop of yeast suspension (OD_600_ = 0.01) was added to a well of a glass bottomed 96 well-plate (MatTek, Ashland, MA). The drop was then scanned to determine the number of cells per drop and the number of foci per yeast cell. Following scanning, drops with single cells were either directly transferred onto ½YPD plates or first grown for 14 h in 200 µl SD medium prior to plating.

### Western Blot Analysis

Cell lysis and Western blotting were performed as described previously with minor modifications [26]. When cells in an actively growing culture reached an OD_600_ of 0.6–0.65 were collected and suspended in an equal volume of lysis buffer (50 mM Tris-HCl, pH 7.4, 200 mM NaCl, 0.5 mM EDTA, pH 8.0, 0.5 mM DTT, and one tablet of a protease inhibitor cocktail (Roche, Indianapolis, IN) in 4 ml volume of buffer, and broken with 0.5 mm glass beads using a mini-bead beater (Biospec Products, Bartlesville OK). After removing cell debris, the total lysate was fractionated by ultracentrifugation at 100,000 rpm for 15 min at 4°C with a TL100 centrifuge (Beckman, CA) and the supernatant was recovered. Identical volumes were loaded on 10% SDS-PAGE gels. Proteins in the gels were transferred to a nitrocellulose membrane (Invitrogen, Carlsbad, CA) by using a wet blotting system (Bio-Rad, Hercules, CA). The following antibodies were used: anti-GFP polyclonal antibody (Abcam), anti-Pgk1 monoclonal antibody (Molecular Probes, Carlsbad, CA) and anti-Hsp104 polyclonal (Abcam). Native Sup35 was detected using a polyclonal rabbit anti-Sup35 antibody made in our laboratory. Hsp70 expression level was detected by monoclonal anti-Hsp70 antibody (Stressgen). Odyssey dye 800-conjugated goat anti-rabbit or donkey anti-mouse antibody was used as secondary antibody. Odyssey scanner and software have been used for detection of bands and quantitation of the intensity of bands on the blots represented in figures.

### Fluorescence Correlation Spectroscopy Measurements and Data Analysis

Fluorescence correlation spectroscopy (FCS) was carried out using the Zeiss ConfoCor system (Thornwood, NY) using an Apochromat 40X/1.2W objective, a 488-nm laser, a 505–550 nm bandpass and a pinhole width of 1 Airy unit. FCS measurements were performed by averaging of 20 consecutive measurements of 1 s for cell measurements and 10 consecutive measurements of 10 s for lysate measurements. Rhodamine 6G (Sigma) was used to calibrate the observation volume. Recombinant GFP (gift G. Patterson, NIH) was used to measure the diffusion coefficient of GFP in solution. For each measurement, a minimum of 10 cells were measured at a given experimental condition. Each experimental condition was repeated a minimum of 5 times and the results of the individual experiments were then averaged. The calculation of the autocorrelation functions was performed during the measurement using the ConfoCor software. The autocorrelation function, *G*(τ) was fitted to the model as follows:
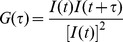
where *I*(*t*+τ) is the fluorescence intensity obtained by the single photon counting method in a detection volume at a delay time τ (brackets denote an ensemble average). The autocorrelation curves were fitted using a one-component fit, i.e. assuming only one class of species, unless specified otherwise. For the fits, the deviation plots showed that the one-component fit was compatible with the data.

## Results

### Curing of [PSI^+^] Prion by Overexpression of Dominant Negative Hsp104 Mutant

**Figure 1:**
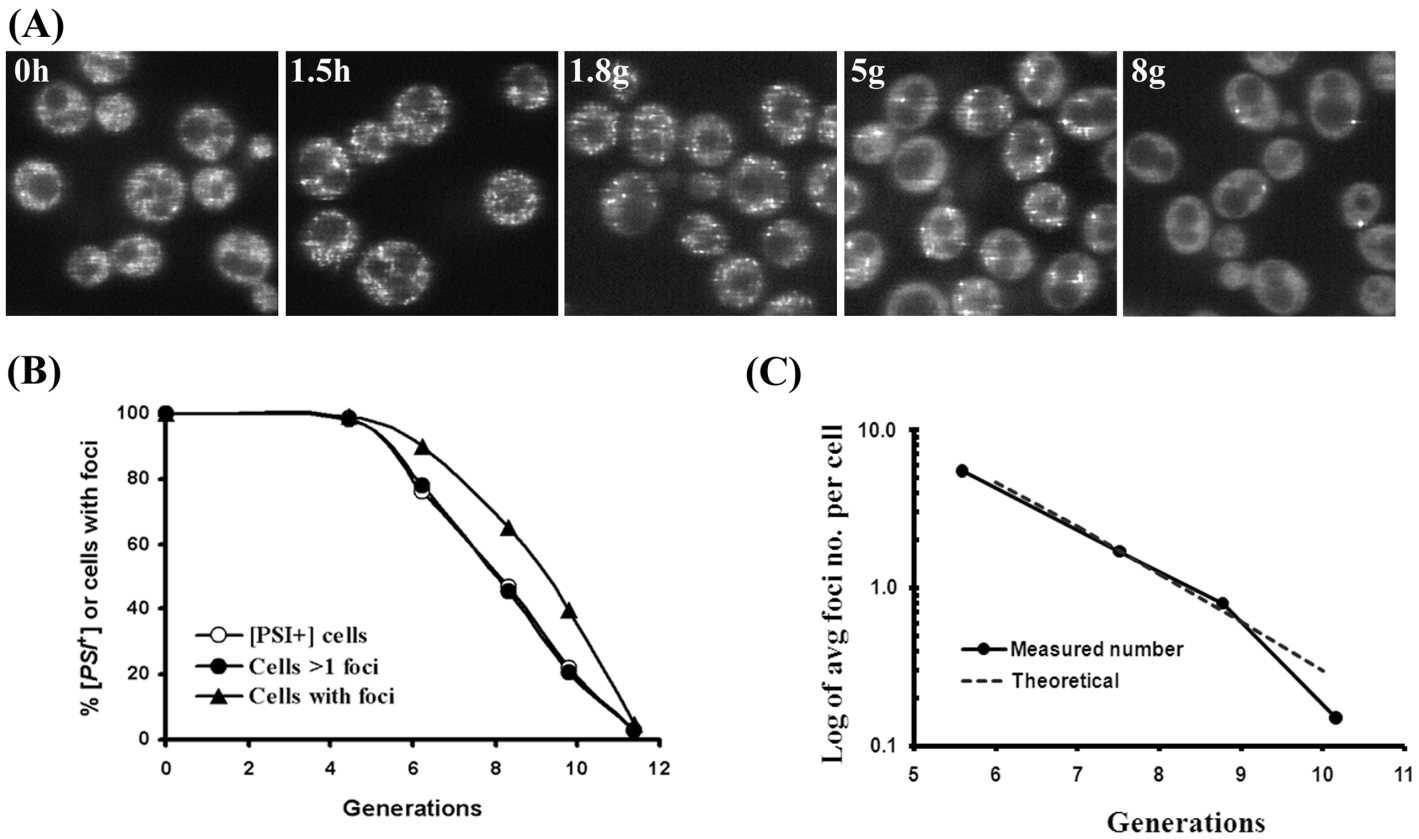
Curing of [*PSI^+^*] by overexpression of Hsp104-2KT. (A) Change in fluorescence of NGMC during curing of [*PSI^+^*] by overexpression of Hsp104-2KT. Cells having pFL39-GAL-HSP104KT grown in SD (0 h) were shifted into SGal and incubated until [*PSI^+^*] seeds were eliminated. Fluorescent images show NGMC in yeast at the indicated growth time (h) or generations (g) from one slice of a Z-stack (16 slides, interval 0.4 µm). (B) Correlation between [*PSI^+^*] cells and cells with foci. Curing of [*PSI^+^*] prion was determined at the indicated generations from the red/white colony count of cells plated on ½YPD plates. The number of cells with foci was counted from Z-stacks of confocal images of fixed cells. Three different experiments yielded similar results. (C) Semi-log plot of average number of foci per cell during curing. For each generation time point, the number of foci was counted in 300–400 cells from Z-stack confocal images of fixed cells. Using the initial data point of 5.5 average foci number at 5.6 generations, the theoretical dashed line was calculated by halving the average foci number at each cell division.

The fluorescence of NGMC was first imaged during the curing of [*PSI^+^*] yeast by overexpression of the Hsp104 dominant negative mutant, Hsp104-2KT. The 1074 yeast strain with *NGMC* integrated into the genomic *SUP35* locus was used to express NGMC at the endogenous Sup35 level. Starting with [*PSI^+^*] yeast, we clearly detected NGMC foci when [*PSI^+^*] yeast were scanned using the Zeiss live confocal microscope. When Hsp104-2KT was induced by galactose, the foci became much more prominent after growing 1.5 h in SGal (Fig. 1A). As noted previously, this change in brightness of the NGMC foci occurred upon changing the medium from SD to SGal, whereas a similar change in media had no effect on the diffusive appearance of NGMC in [*psi^-^*] yeast [27]. This brightening of the fluorescent foci due to the change in medium only lasted several hours until the cells adapted to the new growth condition. Similarly, the foci became more prominent when yeast were incubated in water, which was also reversed upon returning the yeast to SD medium [27].

In contrast to the transient brightening due to the change of medium, the foci remained prominent with continued expression of Hsp104-2KT. As curing occurred, there was a progressive reduction in foci number and a concomitant increase in cytosolic NGMC (Fig. 1A). Ultimately, the NGMC became totally diffusive at which point the yeast were presumably [*psi^-^*]. To determine the relationship between the number of cells with foci and the number of cured cells at different generation times, the yeast cells were either plated to assay prion phenotype by using the red/white colony count or fixed and imaged using Z-stack confocal scanning. From these data, the percentage of cells with foci and the percentage of [*PSI^+^*] cells were plotted as a function of generation time (Fig. 1B). The curves show a similar trend, but at a given generation time, there were more cured cells than cells lacking foci.

Since cells with foci are expected to be [*PSI^+^*], we examined whether the difference between the curves in Fig. 1B was caused by cells with a few foci giving rise to progeny that were [*psi^-^*] on the plate. To test this, Z-stack confocal images of individual cells were obtained following 9 generations of Hsp104-2KT expression. These scanned cells were then plated to determine the prion phenotype. From this analysis, cells without foci (n = 71) yielded red colonies indicating [*psi* ^-^] prion phenotype, while cells with three or more foci (n = 33) yielded white or white-red sectored colonies, indicating [*PSI^+^*] prion phenotype. However, 74% of the cells with only one focus (n = 27) and 27% of the cells with two foci (n = 26) gave rise to red colonies, indicating that the progeny of these [*PSI^+^*] cells were [*psi* ^-^]. This in turn suggested that curing might be occurring on the plate after Hsp104-2KT expression was turned off, but before the remaining Hsp104-2KT was diluted out by cell division, as was proposed by Ferreira et al [15]. Indeed, when cells with a single focus were omitted from the data set, there was excellent agreement between the percentage of cells with more than one focus and the percentage of cells that were [*PSI^+^*] (Fig. 1B, open and closed circles). This suggests that the visible foci are, in fact, the prion seeds with the possible exception of the single focus.

To confirm that the single focus is actually a prion seed rather than a dead end aggregate, individual yeast cells with only one visible NGMC focus were cultured overnight in glucose medium and then plated. We observed that these cells (n = 11) always produced one or more colonies that were either totally white or white-red sectored, which showed that the single focus was indeed a prion seed. In addition to the white or white-red colonies, many of the daughter cells formed red colonies, indicating that inhibition of severing by Hsp104-2KT persisted for several generations until the Hsp104-2KT was finally diluted out. It is this persistence of Hsp104-2KT that causes yeast with a single focus to produce many daughter cells that are [*psi^-^*].

Next, we examined whether the number of fluorescent foci is halved each generation when [*PSI^+^*] was cured by overexpression of Hsp104-2KT as predicted by the cell division model of Tuite and coworkers [16], [18]; this model predicts that inhibition of Hsp104 activity cures prion by inhibiting severing of the prion seeds so that the seeds are diluted out by cell division. To test this prediction, the average number of foci per cell was counted at different generation times during the late stages of prion curing. The semi-log plot of these data (Fig. 1C) shows halving of the average number of foci from generation to generation, in agreement with the results of Cox et al. [28]. These results again indicate that the visible foci are the prion seeds.

The imaging of NGMC foci also provides a method to determine the number of NGMC molecules per focus provided that we can count the number of foci in each yeast cell. Since we could not clearly resolve the individual foci in [*PSI^+^*] cells, the number of foci was counted in [*PSI^+^*] cells after 2 h of Hsp104-2KT induction. At this time the foci had become pronounced, but the yeast had not yet divided in the SGal medium. After fixing the cells, a value of 220±16 (n = 18) foci per cell was counted from the Z-stack confocal images, which is in good agreement with the number of foci that were derived from the curing curve [28]. Knowing the average number of foci per cell, the number of Sup35 molecules in a focus was calculated to be 360, based on the measured value of 80,000 Sup35 molecules per cell [29].

The transmission of foci from mother to daughter was also examined to determine whether the daughter cells inherited 40% of the total foci with the mother cells retaining 60%. This ratio is based on the seeds segregating with equal probability between the mother and daughter cells when corrected for the volume difference between mother and daughter cells [30]. Following overexpression of Hsp104-2KT for 4 and 5 generations, [*PSI^+^*] cells were fixed and bud scars were stained. Daughter cells in the population were identified by the faint staining birth scar. The NGMC foci per cell were counted in daughter cells and in mother cells with either 2 or 3 bud scars. Table 1 shows that the ratio of foci between daughter and mother cells was 2∶3, as predicted by Byrne et al. [30]. In addition, to monitoring the distribution of NGMC foci between mother and daughter cells, live cell imaging was used to follow the transmission of the NGMC foci from mother to daughter cell after expressing Hsp104-2KT for 6–7 generations. Movie S1 shows the transmission of NGMC foci from a mother to a daughter cell, which is representative of more than 20 budding yeast that were imaged. The NGMC foci readily passed from mother to daughter in cells overexpressing Hsp104-2KT, which is consistent with the foci ratio in Table 1. Under these conditions, we do not see a defect in transmission as has been observed in [*PSI^+^*] yeast with active Hsp104 [31].

**Table 1:** Distribution of foci between daughter and mother cells*.

Generations	# of foci Daughter cells^a^	# of foci Mother cells^b^	Ratio (daughter : mother)
4	14 (N = 70)	21.5 (N = 61)	0.42∶ 0.58
5	7.2 (N = 61)	11.6 (N = 71)	0.39∶ 0.61

* Foci were counted in cells having plasmid pFL39-GAL-HSP104KT. The cells were grown for 4 and 5 generations in SGal before to fixing and staining with WGA. N indicates the counted cell number.

a Daughter cells were identified by the faint staining birth scar.

b Mother cells with 2–3 bud scars were used in counting NGMC foci.

### Residual Hsp104 Activity in the Presence of Guanidine

**Figure 2:**
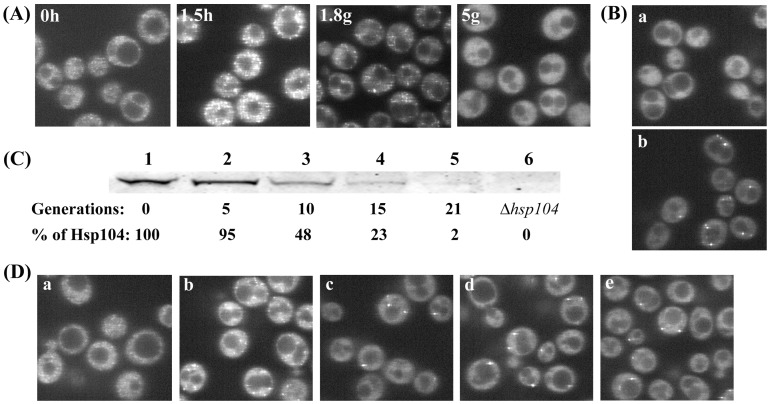
Effect of guanidine on fluorescence of NGMC foci in [*PSI^+^*] yeast in presence and absence of Hsp104. (A) Change in fluorescence of NGMC during curing by guanidine. Cells were grown in SD with 5 mM guanidine for the indicated growth time in hours (h) or generations (g). (B) Fluorescent imaging of [*PSI* ^+^] cells either with the empty vector (a) or plasmid pFL39-GAL-HSP104KT expressing Hsp104-2KT (b) were grown for 5 generations in SGal with 5 mM guanidine. (C) Western blot analysis of Hsp104 to determine the level of Hsp104 following excision of Hsp104 after inducing expression of Flp recombinase. Yeast were grown in SGal to induce expression of Flp recombinase to delete the *FRT*-sites flanked *HSP104.* Lysates were obtained from the conditional deletion yeast strain of *HSP104* grown in SD (lane 1) and SGal for indicated generations (lane2–5), and the Δ*hsp104* strain (lane 6). The percent Hsp104 calculated from the intensity at each time relative to the intensity of the control was corrected for protein loading using the Pgk1 standard. (D) Fluorescent microscopic images of yeast expressing NGMC before and after conditional deletion of *HSP104*. The conditional deletion yeast strain of *HSP104* was grown in SD (a) or in SGal for 10 generations (b), followed by another 5 generations in SGal with (c) and without (d) 5 mM guanidine. Cells in panel (d) were incubated for 1 h in water (e). Images show one slice from a Z-stack (16 slides, interval 0.4 µm).

Although the above data establish that the visible foci are the prion seeds, this conclusion appears to contradict our previous result that when [*PSI^+^*] was cured by guanidine, the NGMC fluorescence became diffusive even though the cells were not yet cured [21]. These earlier results are confirmed by the images in Fig. 2A, which show the fluorescence of NGMC in yeast during curing of [*PSI^+^*] by guanidine. In agreement with our previous study [21], after addition of guanidine the foci first became brighter and then disappeared so that after 3–4 generations, the NGMC fluorescence appeared completely diffusive in all of the cells, indistinguishable from the fluorescence of [*psi^-^*] yeast even though the cells were all [*PSI^+^*] based on the colorimetric colony assay. Interestingly, if curing of [*PSI^+^*] were done in SGal medium to induce expression of Hsp104-2KT, as well as 5 mM guanidine, the NGMC foci remained bright, but this did not occur in control cells with an empty vector when these cells were cured in SGal medium with 5 mM guanidine (Fig. 2B). Based on these results, we conclude that the loss of detectable foci during curing of [*PSI^+^*] yeast by guanidine is blocked by overexpression of Hsp104-2KT.

Since foci were prominent during curing of [*PSI^+^*] yeast by Hsp104-2KT, but not by guanidine treatment, we wanted to determine whether prominent NGMC foci also occurred in the absence of Hsp104. To examine the effect of Hsp104 depletion on NGMC fluorescence, [*PSI^+^*] yeast were cured by conditionally deleting the *HSP104* gene using the FLP/FRT recombination system. For this experiment, we used a Δ*hsp10*4 strain of [*PSI^+^*] yeast transformed with pRS315-FRT-HSP104 and pRS314-GAL1-FLP plasmids (see Materials and Methods). When these yeast were grown in SGal medium to induce expression of Flp recombinase, this produced excision of the *HSP104* gene. Western blot analysis showed that the level of Hsp104 in the yeast population was 48% and 23% of the control value after 10 and 15 generations in SGal medium, respectively, and by 21 generations, essentially all of the Hsp104 protein was depleted from the yeast cells (Fig. 2C). The fact that the level of Hsp104 is not halving each generation indicates that *HSP104* excision is occurring over many generations in the yeast population.

Fluorescent imaging of the yeast cells showed that after 10 generations in SGal medium to induce Flp recombinase expression, more than 90% of the cells still had prominent foci (Fig. 2D) and with further divisions, the number of foci per cell decreased and gradually all of the NGMC became diffusive, characteristic of [*psi* ^-^]. Therefore, depletion of Hsp104 produces a similar effect on the NGMC foci as expression of Hsp104-2KT. Furthermore, when cells were first grown for 10 generations in SGal medium to excise *HSP104* and then incubated further for another 5 generations with guanidine, the remaining foci still appeared very prominent (Fig. 2D, panel c), no different from cells grown in the absence of guanidine (Fig. 2D, panel d). Furthermore, there was no significant change in the number of cells with foci or the foci intensity when the cells were placed in water for 1 h (Fig. 2D, panel e). Therefore, the NGMC foci do not become diffusive in the presence of guanidine in the absence of Hsp104, which confirms that Hsp104 is responsible for the loss of detectable NGMC foci during curing of [*PSI^+^*] by guanidine.

### Relationship between the Foci and the Seeds During Curing by Guanidine

**Figure 3:**
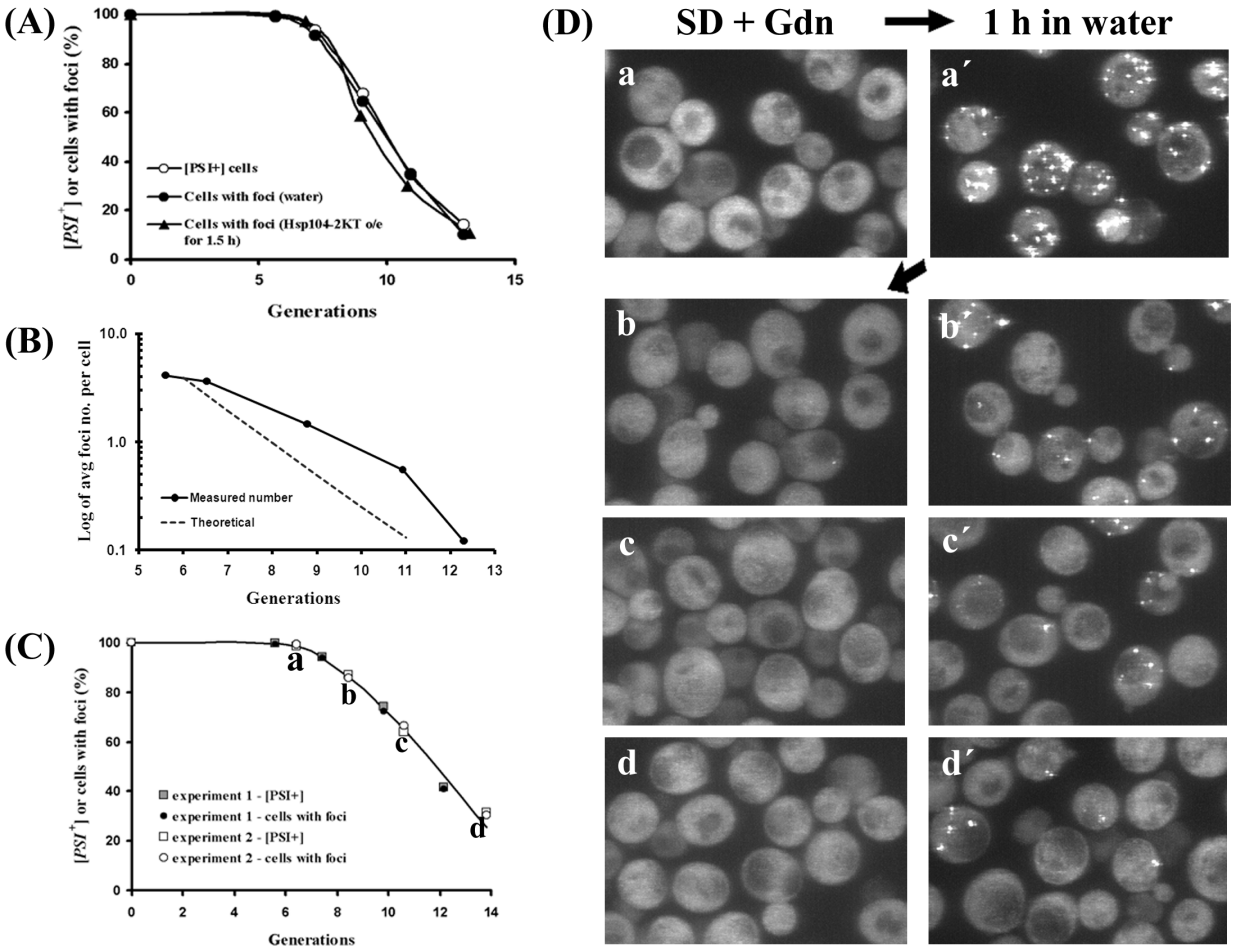
Curing of [*PSI^+^*] by guanidine. (A) Correlation between cells with foci and [*PSI* ^+^] yeast as a function of generation time during curing by guanidine. The kinetics of [*PSI* ^+^] curing was measured by plating yeast at indicated generations on ½YPD plates. Yeast having plasmid pFL39-GAL-HSP104KT were grown in SD with 5 mM guanidine. To visualize the foci during curing, cells were incubated at indicated generations either in SGal for 1.5 h to express of Hsp104-2KT or in water for 1 h. The number of cells with foci was counted from Z-stacks of confocal images of fixed cells. Three different experiments yielded similar results. (B) Semi-log plot of average number of foci per cell during curing of [*PSI^+^*] by guanidine. For each generation time point, the number of foci was counted in 300–400 fixed cells from Z-stacks of confocal images of fixed cells. Using the initial data point of 4.1 average foci number at 5.6 generations, the theoretical dashed line was calculated by halving the average foci number at each cell division. (C) Curing and fluorescent images of NGMC in yeast during final stages of curing by guanidine. [*PSI^+^*] yeast grown for ca. 5 generations in SD with 5 mM guanidine were incubated in water for 1 h to visualize foci, and then the yeast were returned to SD with 5 mM guanidine and incubated further for 7–8 generations. At indicated generations, cells were incubated in water for 1 h and imaged by Z-stack confocal microscopy. Two different experiments are shown. (D) Imaging of the NGMC foci during the late stages of curing of [*PSI^+^*] by guanidine. The maximum Z-stack projections of the yeast cells are shown before (a-d) and after incubation in water for 1 h (a′-d′). Cells were imaged in experiment #2 in (C), where a-d shows the indicated generation.

Since the foci are made prominent during curing of [*PSI^+^*] by guanidine by transient incubation in water or in SGal to induce expression of Hsp104-2KT, we used these methods to further confirm that the foci are the seeds. At different time points during [*PSI^+^*] curing by guanidine, aliquots of cells were plated to determine the prion phenotype, while other aliquots were grown in SGal medium for 1.5 h to induce Hsp104-2KT. As shown in Fig. 3A, the analysis of colony color and the fluorescent images showed that the percentage of cells that are [*PSI^+^*] and the percentage of cells with foci were essentially identical. The same results were obtained when the partially cured cells were placed in water for 1 h prior to imaging to increase the intensity of the foci.

In contrast to curing by overexpression of Hsp104-2KT, there was excellent agreement between the percent [*PSI^+^*] cells and the percent cells with foci without having to correct the data for cells with a single focus since guanidine is immediately removed from the yeast upon plating. This was confirmed by determining that the cells with a single focus had a [*PSI^+^*] phenotype after plating. Specifically, after growing [*PSI^+^*] cells for 9 generations with guanidine, the cells were placed in water for 1 h. Z-stack scanning and the plating of individual cells showed that cells without foci (n = 36) formed red colonies, while cells with one (n = 15) or more foci (n = 17) formed white or occasionally white-red sectored colonies. Therefore, the foci are indeed the prion seeds, although the seeds do not always have to be visible as foci.

Previously the absence of visible foci in yeast incubated with guanidine led us to incorrectly propose that when cells were cured by guanidine, the number of seeds was actually reduced without cell division [21]. Experiments in which cell division was blocked by alpha factor seemed to corroborate this conclusion since when cells were grown with alpha factor for more than one day, cured cells appeared. In fact, it turned out that our results were caused by the combination of cells resistant to alpha factor that continued to divide in the presence of alpha factor and toxicity caused by alpha factor in the non resistant cells [18]. Therefore, although the foci are not detectable during curing by guanidine, only cell division actually reduces the number of seeds.

We next examined whether the number of seeds halved each generation during the curing of [*PSI^+^*] by guanidine. After growing the yeast for 5 or more generations in the presence of guanidine, the yeast were starved for 1 h in water prior to fixation to enable detection of the foci. By imaging confocal Z-stacks of the fixed cells, we determined the average number of foci as a function of generation time. In agreement with Cox et al. [28], the number of foci decreased with increasing generation time, but as shown by semi-log plot of average foci number per cell versus generation time, the data deviated from the theoretical line calculated on the basis of the foci halving each generation (Fig. 3B). As pointed out by Byrne et al [18], this deviation could be due to guanidine not completely inhibiting the severing activity of Hsp104. In fact, the deviation from the theoretical line is consistent with 20% of the existing seeds being severed at each generation. This amount of severing would also account for the observation that the curing of [*PSI^+^*] by guanidine lags by 2–3 generations compared to the curing by overexpression of Hsp104-2KT. Therefore, Hsp104 appears to retain some residual severing activity in 5 mM guanidine, in contrast to the complete inhibition of severing activity observed upon overexpression of Hsp104-2KT.

To further check that this residual severing activity does not account for the loss of detectable foci during curing by guanidine, we repeatedly imaged the foci during the final stages of curing. After 5 generations of growing [*PSI^+^*] yeast with guanidine, at which time the cells all showed diffuse fluorescence but still were [*PSI^+^*], the yeast were incubated in water for 1 h to make the foci prominent. The yeast were returned to SD medium with guanidine, which again caused diffuse fluorescence and further curing. At different time points during the curing cycle, the cells were incubated in water for 1 h, followed by Z-stack confocal microscopy. The maximum Z-stack projections of the yeast cells imaged at indicated generations in Fig. 3D show a marked reduction in the number of foci. Along with this reduction in foci number, there is, as expected, a reduction in the number of cells with foci (Fig. 3C). Importantly, severing could only cause all of the seeds to become undetectable if all of the seeds, not just 20% were severed. If this were the case, then there would not have been a steady reduction in foci number as the cells divided. The fact that the number of seeds markedly decreased shows that severing is not causing the loss of detectable foci. Therefore these results confirm that the loss of detectable foci is not due to the residual severing activity of Hsp104.

### Factors that Affect the Fluorescence of NGMC Foci during Curing of [*PSI^+^*]

**Figure 4:**
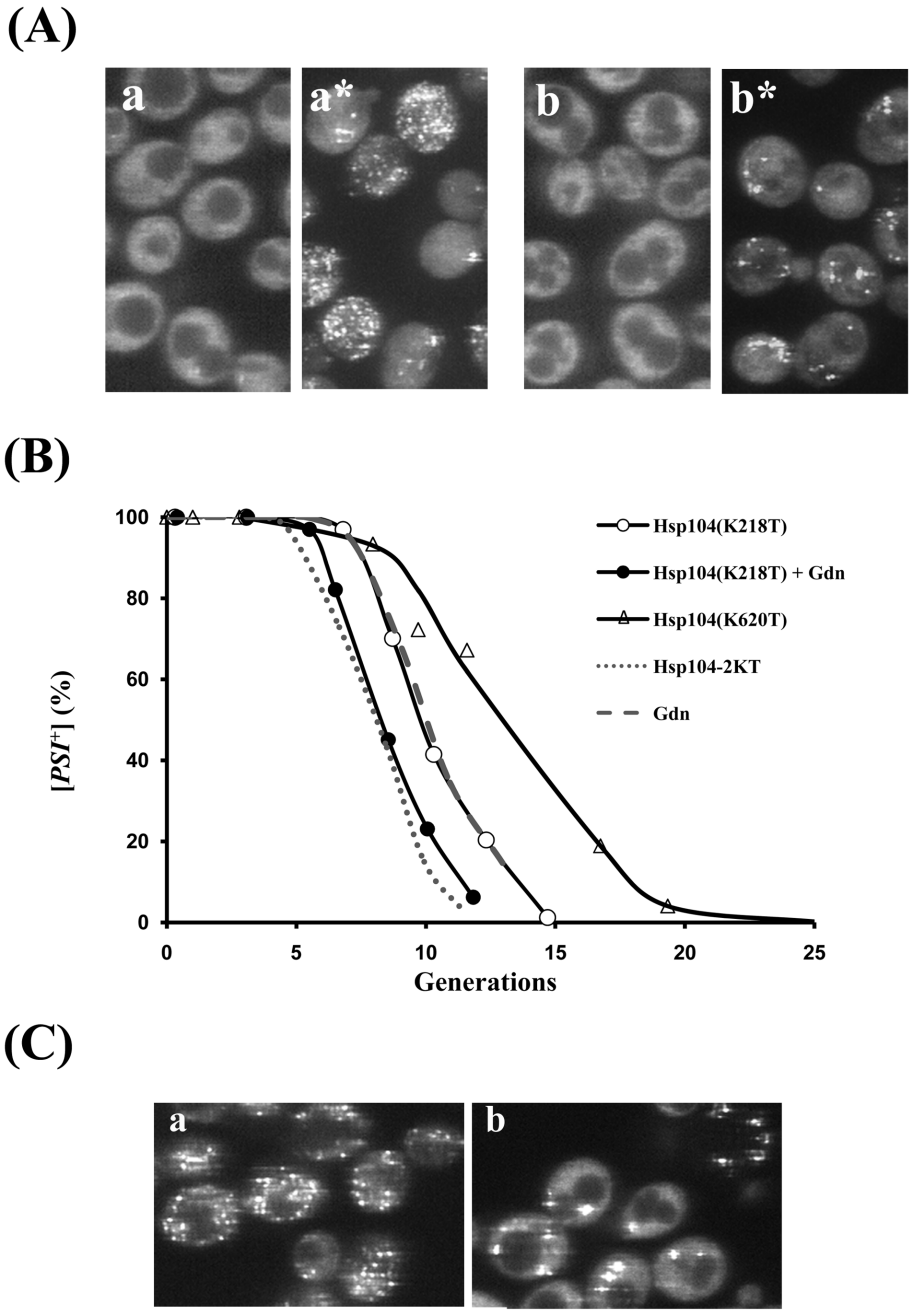
Curing of [*PSI^+^*] by overexpression of either Hsp104(K218T) or Hsp104(K620T). (A) Fluorescent images of NGMC were from partially cured [*PSI^+^*] yeast grown in SGal to induce overexpression of Hsp104(K218T) for 7.5 generations (a), followed by incubation in water for 1 h (a*). Fluorescent images of NGMC were from yeast grown for 5.5 generations in SGal to induce overexpression of Hsp104(K218T) in the presence of 5 mM guanidine (Gdn) (b), followed by incubation in water for 1 h in water (b*). (B) Kinetics of [*PSI* ^+^] curing by overexpression of Hsp104(K218T) and by overexpression of Hsp104(K620T). The curing of [*PSI* ^+^] either by overexpression of Hsp104(K218T) without (open circles) and with guanidine (closed triangles) or by overexpression of Hsp104(K620T) (closed circles) was plotted as a function of generation time. For comparison, the curing curves of [*PSI* ^+^] strain by overexpression of Hsp104-2KT and by guanidine are shown by the dotted and dashed lines, respectively. (C) Fluorescent images of [*PSI^+^*] yeast partially cured by overexpression of Hsp104(K620T) for 3 and 9 generations.

The Hsp104-2KT mutant has point mutations at the active sites of both NBD1 (K218T) and NBD2 (K620T), whereas it has been suggested that guanidine inhibits the activity of just the NBD1 domain [32]. This led us to specifically inactivate the NBD1 domain of Hsp104; biochemical studies showed that mutating the active site of NBD1 essentially eliminated the ATPase activity of Hsp104, but not the hexamerization of the Hsp104 subunits [33]. Interestingly, when [*PSI^+^*] was cured by overexpression of Hsp104(K218T), just as occurred during curing by guanidine, the NGMC foci became undetectable in the majority of the cells even though the cells were still [*PSI^+^*] (Fig. 4A). Along with the loss of detectable foci, the rate of curing by overexpression of Hsp104(K218T) was slightly delayed compared to the rate of curing by overexpression of the Hsp104-2KT. The time course of curing by overexpression of Hsp104(K218T) was the same as that observed during curing by guanidine. These results show that that Hsp104(K218T) has limited severing activity, which in turn shows that the NBD2 domain of Hsp104 also contributes to the severing activity of Hsp104. Therefore, the Hsp104(K218T) mutant has markedly reduced severing activity but retains a residual activity that apparently leads to the loss of detectable foci, similar to the effect of guanidine treatment on Hsp104 activity Evidently, complete inhibition of Hsp104 activity requires mutating the active sites of both NBD1 and NBD2.

If guanidine is curing solely by inactivating the NBD1 domain of Hsp104, then guanidine should have no effect on the curing of [*PSI^+^*] by overexpression of Hsp104(K218T). However, addition of guanidine caused faster curing of [*PSI^+^*] by Hsp104(K218T) (Fig. 4B). Specifically, the rate of curing increased by about 2 generations, which shows that guanidine is not just inactivating the NBD1 domain. In fact, the time course of [*PSI^+^*] curing by the combination of overexpression of Hsp104(K218T) and 5 mM guanidine was essentially the same as that observed when [*PSI^+^*] was cured by overexpression of Hsp104-2KT. Therefore, this combination essentially eliminated the severing activity of Hsp104. At the same time, NGMC foci were not visible in cells that were still [*PSI^+^*] (Fig. 4A), which results rule out that limited severing activity produces the loss of detectable NGMC foci.

The above results predict that the NBD2 domain of Hsp104 is acting to make the NGMC foci undetectable during curing. To test this prediction, we expressed Hsp104(K620T), which contains a point mutation only at the active site of the NBD2 domain of Hsp104. After inducing Hsp104(K620T) expression by galactose in [*PSI^+^*] yeast, the cells were imaged and plated on ½ YPD plates. In agreement with the prediction, prominent foci were still present both at 3 and 8 generations after induction of Hsp104(K620T) (Fig. 4C). Fig. 4B shows that overexpression of Hsp104(K620T) cured [*PSI^+^*] yeast, but at a much slower rate than overexpression of either Hsp104-2KT or Hsp104(K218T). Therefore, the NBD2 domain, as well as the NBD1 domain of Hsp104, functions in the severing activity of Hsp104, but the NBD1 domain appears to have a greater severing activity than the NBD1 domain. This difference in severing activity between the NBD domains of Hsp104 has the caveat that there may be less hexameric Hsp104(K620T) compared to Hsp104(K218T) in the yeast cytosol based on in vitro studies showing that the Hsp104(K620T) mutant has impaired oligomerization [33].

**Figure 5:**
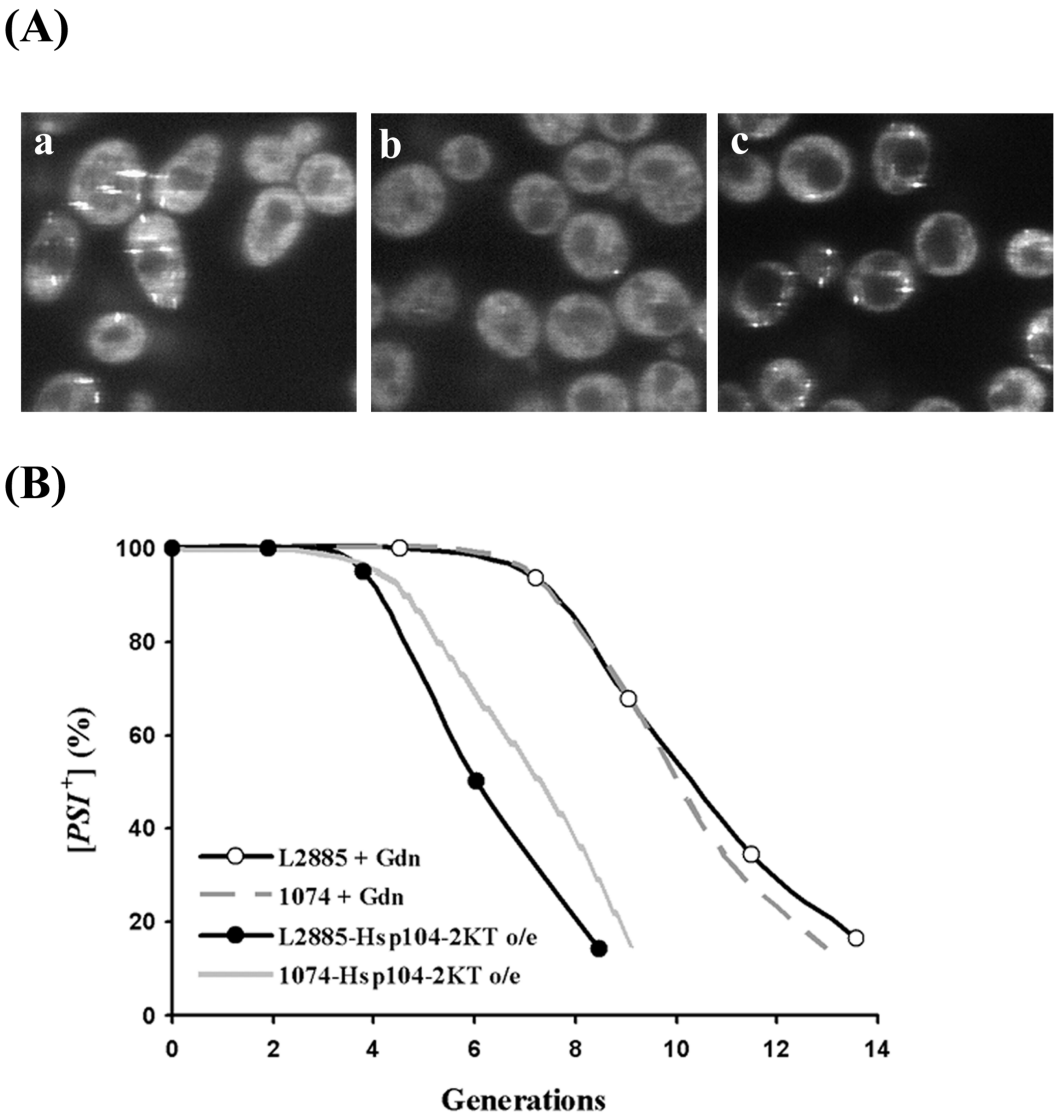
Curing of [*PSI^+^*] by overexpression of Hsp104-2KT or by guanidine in strain L2885. (A) Fluorescence images of NGMC were from partially cured L2885 [*PSI^+^*] yeast grown for 6 generations either in SGal to induce overexpression of Hsp104-2KT (a) or in SD with 5 mM guanidine (b) followed by 1 h incubation in water (c). (B) Kinetics of [*PSI* ^+^] curing by Hsp104 inactivation in strain L2885. The curing of [*PSI* ^+^] by overexpression of Hsp104-2KT (closed circles) or by guanidine (open circles) is plotted as a function of generations. For comparison, the curing curves of [*PSI* ^+^] strain by overexpression of Hsp104-2KT and by guanidine in strain 1074 are shown by the gray line and the gray dashed line, respectively.

We also examined whether the decrease in fluorescence intensity of the seeds during curing of [*PSI^+^*] by guanidine is dependent on the yeast strain by using the L2885 yeast strain derived from the wild-type strain 74-D694 [22]. This strain expresses NGMC from the *SUP35* genomic locus using the NGMC construct engineered in the Serio laboratory [13]. Both the change in fluorescence and the prion phenotype were monitored in the L2885 strain during curing of [*PSI^+^*] either by overexpression of Hsp104-2KT or by guanidine treatment. With this strain, there were also prominent foci in cells grown for 6 generations in SGal to overexpress Hsp104-2KT as compared to the absence of detectable foci in yeast grown in 5 mM guanidine (Fig. 5A). Under the latter conditions, the foci again became visible when the cells were incubated in water (Fig. 5A, panel c). Fig. 5B shows that the kinetics of [*PSI^+^*] curing in the L2885 strain when Hsp104 was inactivated either by overexpression of Hsp104-2KT or by guanidine were similar to the kinetics of [*PSI^+^*] curing in the 1074 strain. Therefore, regardless of the yeast strain, there is a decrease in the fluorescence intensity of the seeds when [*PSI^+^*] is cured by guanidine as opposed to overexpression of Hsp104-2KT.

**Figure 6:**
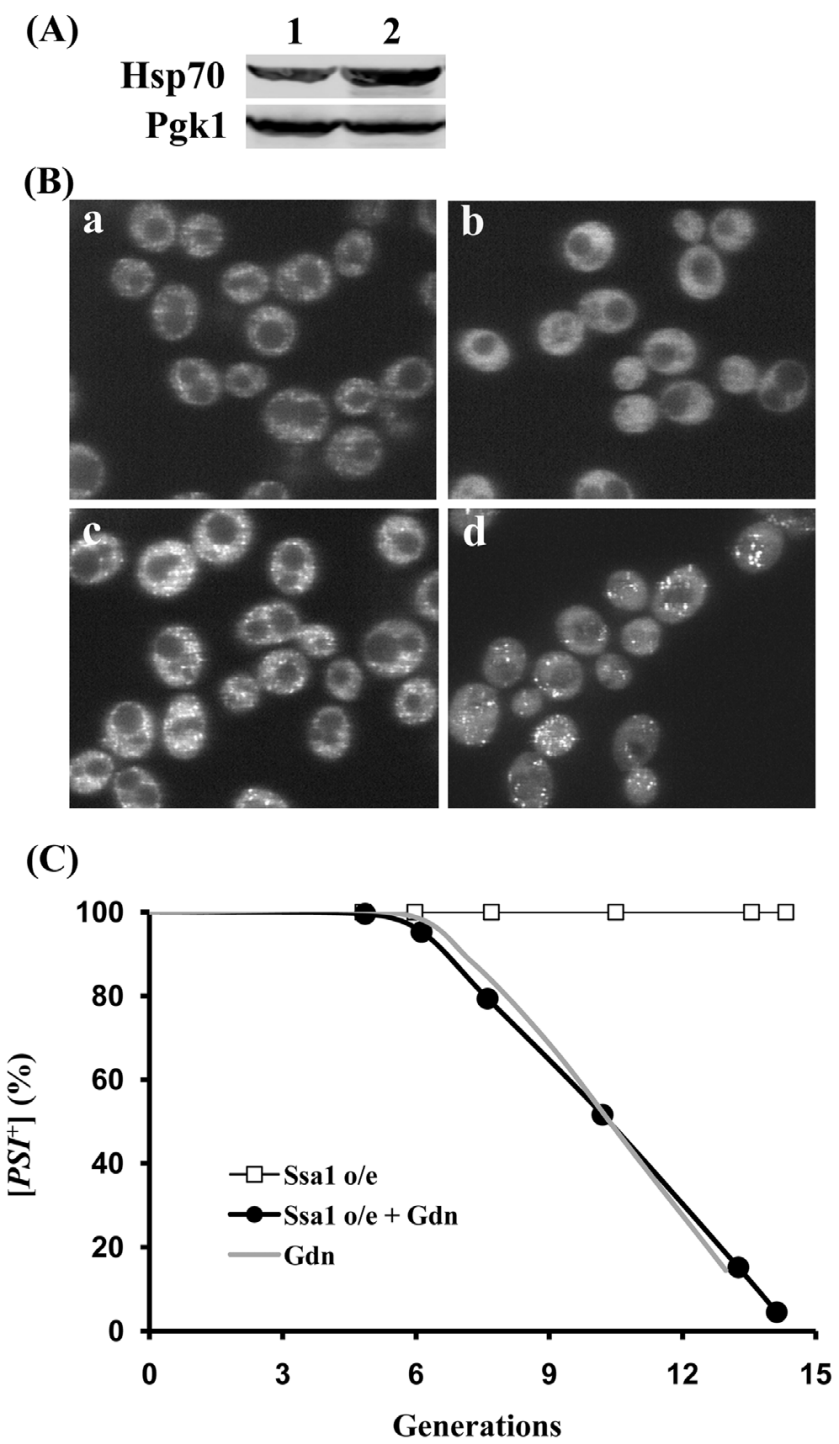
Effect of overexpression of Ssa1 on NGMC fluorescence and prion propagation in [*PSI* ^+^] yeast in the presence and absence of guanidine. (A) Western blot analysis of Hsp70 expression levels. The lanes are as follows: lane 1; 1074 with pRS314-GAL1-SSA1 in SD, lane 2; 1074 with pRS314-GAL1-SSA1 in SGal for 5 generations. The Pgk1 probed by anti-Pgk1 antibody on the same blot was used as an internal loading control. (B) Effect of overexpression of Ssa1 on NGMC fluorescence. Cells of strain 1074 having the empty vector pRS314 were grown in SGal for 5 generations without (a) and with 5 mM guanidine (b). Cells having pRS314-GAL1-SSA1 were grown under the same conditions for 5 generations without (c) and with 5 mM guanidine (d). (C) Effect of overexpression (o/e) of Ssa1 on [*PSI* ^+^] propagation and curing by guanidine (Gdn). [*PSI* ^+^] yeast containing pRS314-GAL1-SSA1 were incubated in SGal in the absence (open squares) or presence of 5 mM guanidine (solid circles). The dotted line shows the curing of [*PSI* ^+^] yeast with empty vector incubated in SGal with 5 mM guanidine.

Since Ssa1 has been shown to antagonize the action of Hsp104 [34], we examined the effect of Ssa1 overexpression on the curing of [*PSI* ^+^] by guanidine. Ssa1 under the control of the *GAL1* promoter was expressed by growing yeast in SGal medium, which doubled the expression level of Ssa1 (Fig. 6A). Compared to the empty vector controls grown in SGal medium (Fig. 6B, panel a), overexpression of Ssa1 caused an increase in the brightness of the NGMC foci in [*PSI* ^+^] yeast (Fig. 6B, panel c), as shown previously with the L2885 strain [22]. However, in contrast to the L2885 strain, overexpression of Ssa1 did not cure [*PSI* ^+^] in the 1074 yeast strain even when expressed for 20 generations (Fig. 6C), indicating that severing is not inhibited. Imaging of the NGMC fluorescence in yeast overexpressing Ssa1 showed marked variability in the brightness of the foci possibly due to severing creating a broad size distribution of seeds. When Ssa1 was overexpressed during the curing of [*PSI* ^+^] by guanidine treatment, the NGMC foci did not become undetectable, but rather were clearly visible until the yeast were cured (Fig. 6B, panel d), whereas the empty vector control did not show detectable foci (Fig. 6B,b). Even though the NGMC foci were visible until the cells were cured, overexpression of Ssa1 did not effect on the kinetics of [*PSI* ^+^] curing (Fig. 6C), which again indicates that Ssa1 does not affect severing. These observations suggest that increased expression of Ssa1 antagonizes the activity of Hsp104 that renders the foci undetectable by fluorescence imaging during the curing of [*PSI* ^+^] by guanidine treatment.

### Diffusional Mobility of NGMC in [*psi^-^*], [*PSI^+^*] and Partially Cured [*PSI^+^*] Yeast

**Figure 7:**
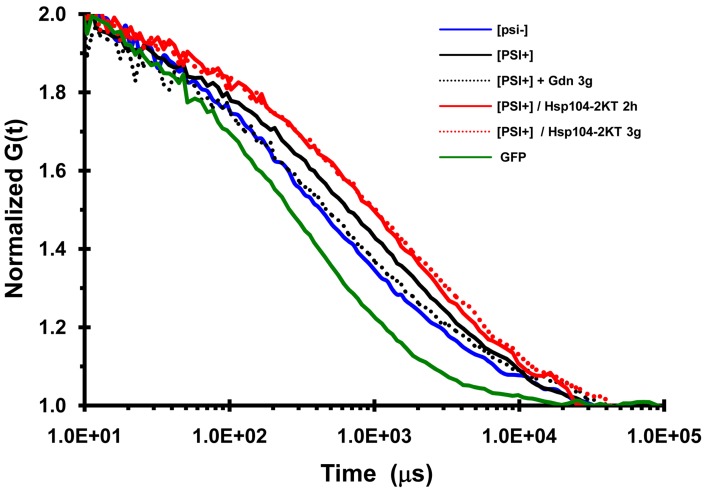
Autocorrelation curves of NGMC in yeast. FCS measurements are of GFP in [*PSI* ^+^] yeast, NGMC in [*psi* ^−^] cells, NGMC in [*PSI* ^+^] cells, and NGMC in [*PSI* ^+^] cells grown in SGal either for 2 h or 3 generations (g) to overexpress Hsp104-2KT, and NGMC in [*PSI* ^+^] cells grown for 3 generations in SD with 5 mM guanidine (Gdn). Since measurements of the GFP diffusional mobility was not significant different between [*psi* ^−^] and [*PSI^+^*] cells, only the curve obtained with [*PSI* ^+^] cells is shown.

To determine whether the size of the NGMC foci are reduced during the curing of [*PSI* ^+^] by guanidine, as suggested by the observation that the NGMC foci became undetectable, we used FCS to measure the diffusional mobility of the seeds. First, we measured the diffusional mobility of NGMC in [*psi^-^*] and [*PSI^+^*] yeast; as a control we also measured the diffusional mobility of GFP (Fig. 7). As expected, the autocorrelation curves showed that NGMC had a much slower mobility in [*PSI^+^*] than in [*psi^-^*] yeast. In addition, the diffusional mobility of NGMC became slower and quite heterogeneous when Ssa1 was overexpressed. To analyze the data more quantitatively, the autocorrelation curves were fitted with a 1-component fit since essentially all of the Sup35 is soluble in [*psi^-^*] yeast and aggregated in [*PSI* ^+^] yeast. Both in [*psi^-^*] and [*PSI^+^*] yeast, GFP had a dwell time of about 0.3 msec, which was used to calculate a diffusion coefficient of 22 µm^2^s^−1^ [35], in good agreement with values from other laboratories [36], [37]. NGMC had dwell time of about 1.0 msec in [*psi^-^*] yeast, as shown previously [20], whereas the dwell time of NGMC was 2.5 msec in [*PSI* ^+^] yeast.

Given the molecular weight of GFP, apparent molecular weights were calculated using the proportionality that the molecular weight scales with the cube of the dwell time for globular proteins. On the basis, NGMC has an apparent molecular weight of about 1000 kDa in [*psi^-^*] yeast, which is much greater than the molecular weight of Sup35, even if it forms a 1∶1 complex with Sup45. This high molecular weight is consistent with Sup35 binding to ribosomes. As for the apparent molecular weight of NGMC in [*PSI* ^+^] yeast, a value of about 14,000 kDa was determined. Based on one molecule of Ssa1/SSa2 binding to 2 molecules of Sup35 in the amyloid complex [38], this implies that there are approximately 100 molecules of NGMC per seed in [*PSI* ^+^] yeast.

We also measured the diffusional mobility of the NGMC foci after 2 h of Hsp104-2KT induction, the same condition used above to count the number of NGMC foci. As shown in Fig. 7, NGMC had a slower mobility following induction of Hsp104-2KT than it does in [*PSI^+^*] yeast, consistent with the brighter NGMC foci observed in cells expressing Hsp104-2KT. A value of about 5 msec was obtained by fitting the autocorrelation curve, indicating that each focus has roughly 800 NGMC molecules per seed calculated from the molecular weight of NGMC and assuming that there is one molecule of Ssa1/Ssa1 bound to Sup35 in the amyloid fiber. This value of 800 molecules per seed is about twice the value of 360 NGMC molecules per seed that was calculated above by counting the number of foci per cell and the value of 80,000 molecules of Sup35 per cell [29]. The approximate 2-fold difference in mass may be due to other proteins, in addition to Ssa1/Ssa2, binding to the amyloid fibers as well as the assumption, used to calculate the mass that the amyloid fiber had a globular shape.

Next we compared the mobility of NGMC during curing of [*PSI^+^*] yeast by overexpression of Hsp104-2KT and curing by guanidine treatment. This comparison was made at 3 generations, the earliest time at which the foci became undetectable in the majority of [*PSI^+^*] cells during curing by guanidine treatment. Fig. 7 shows that the autocorrelation curves of NGMC were very different for the two conditions. Specifically, the mobility of NGMC in yeast overexpressing Hsp104-2KT was slower than in [*PSI* ^+^] yeast, while the mobility of NGMC in guanidine-treated yeast was faster than in [*PSI* ^+^] yeast. In fact, the mobility of NGMC in guanidine-treated yeast is essentially the same as that of NGMC in [*psi^-^*] yeast.

To calculate the diffusional mobility of the seeds under these various conditions, we again analyzed the data using a 1-component fit for the autocorrelation curves. In lysates from [*PSI* ^+^] yeast expressing Hsp104-2KT for 3 generations, there was no increase in the soluble NGMC fraction compared to lysates form [*PSI^+^*] yeast as determined by Western blot analysis (Table 2). This indicates that essentially all of NGMC is aggregated. Fitting of the autocorrelation curves for NGMC in yeast expressing Hsp104-2KT for 3 generations yielded a dwell time of about 6 msec, which indicates that each focus has roughly 1400 NGMC molecules per seed calculated from the molecular weight of NGMC and the assumption that one Ssa1/Ssa2 molecule bound per 2 Sup35 molecules in the amyloid fiber. This increase in the size of the seeds from 800 Sup35 molecules per seed to 1600 molecules per seed between 2 and 3 generations is consistent with the observed increase in brightness and suggests that newly synthesized NGMC bound to the seeds rather than becoming soluble.

**Table 2:** Soluble Sup35 in [*PSI* ^+^] yeast expressing Hsp104-2KT.

	[*PSI* ^+^]	Hsp104-2KT		
Generations		1.8	3.4	5
**NGMC**	6.3	3.7	5.7	10.1
**Pgk1**	28.6	20.7	25.1	27.9
**fraction NGMC** b	1.0	0.6	0.9	1.6
**corrected fraction NGMC** c	1.0	0.8	1.0	1.7

a Protein intensity of NGMC was quantified using the Odyssey software. Yeast were cured by overexpression of Hsp104-2KT for the indicated generations. The fractions were also probed with Pgk1p as a loading control.

b Fraction of NGMC was calculated by normalizing the data to the soluble NGMC in [*PSI* ^+^] yeast.

c Fraction of NGMC was corrected for Pgk1 levels.

As for determining the diffusional mobility of the seeds in [*PSI* ^+^] yeast treated with guanidine for 3 generations, the data were first fitted using a 1-component fit, which yielded a dwell time of about 1 msec, indicating a molecular weight of 1000 Kda for the NGMC complex. These same data were also fitted using a 2-component fit since the yeast cytosol not only contains soluble NGMC, but also seeds, which became visible upon transient expression of Hsp104-2KT or incubation of the yeast in water. In fitting the data using a 2-component fit, the dwell time of the fast component was fixed at 1 msec, the value for soluble NGMC measured in [*psi^-^*] yeast. On this basis, more than 90% of the NGMC had a mobility of 1 msec, while the slower component had a dwell time of about 10 msec, indicating a molecular weight for the NGMC complex of 900,000 kDa. Given that the partially cured [*PSI* ^+^] yeast have more than a 100 seeds based on the kinetics of curing [18], it is not possible for this high molecular weight component represents the seeds. Instead, the high molecular weight component appears to be an immobilized NGMC fraction, which was also previously observed [20], is also present in [*psi^-^*] yeast when the autocorrelation curve of NGMC was fitted to a 2-component fit with the same parameters. These results indicate that the seeds in guanidine-treated [*PSI* ^+^] yeast are in the same fraction as the soluble material and therefore, have the same molecular weight as the soluble material. This in turn implies that there are approximately 8 NGMC molecules per seed. Therefore, NGMC seeds are much smaller in guanidine-treated [*PSI^+^*] yeast than in yeast cured by overexpression of Hsp104-2KT.

### *In vitro* Studies Measuring the Fraction of NGMC Monomer in Partially Cured [PSI^+^] Yeast

**Figure 8:**
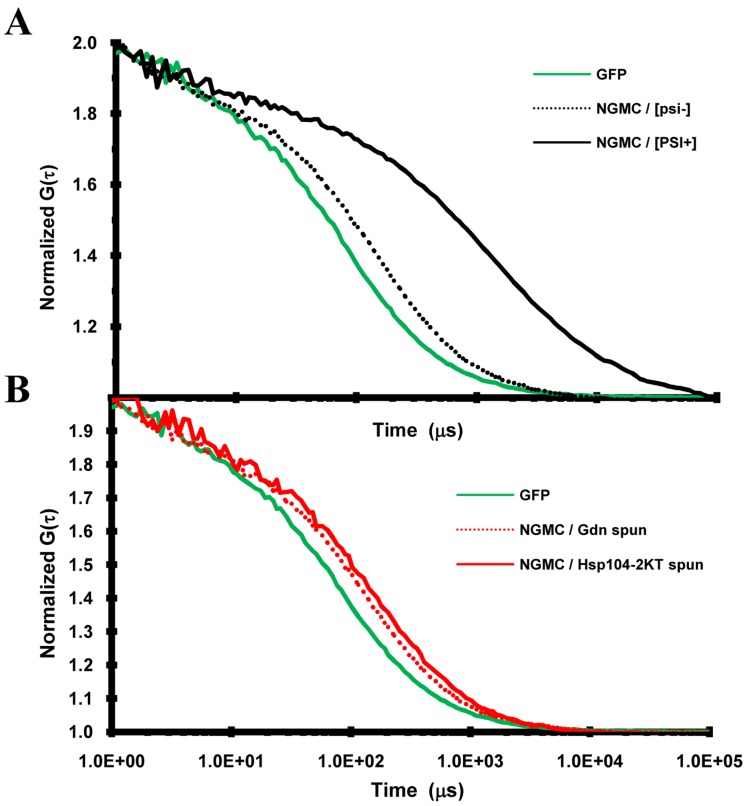
Autocorrelation curves of NGMC in yeast lysates. (A)Diffusion mobility of GFP and NGMC. FCS measurements are of GFP in phosphate buffered saline and of NGMC in lysates from [*psi* ^−^] and [*PSI* ^+^] yeast, (B) Diffusional mobility of NGMC was measured in the supernatant from lysates of [*PSI* ^+^] yeast following high speed centrifugation. Lysates were prepared from yeast grown for 5 generations either in SD with 5 mM guanidine (Gdn) or in SGal to overexpress Hsp104-2KT. GFP standard is included in this graph.

All of the above studies used live cell imaging to study the properties of NGMC during the curing of [*PSI^+^*] yeast by inactivation of Hsp104. To further characterize the difference in prion size during curing by guanidine treatment or by overexpression of Hsp104-2KT, we examined the properties of NGMC in yeast lysates prepared from [*PSI^+^*], partially cured, and [*psi^-^*] yeast. If the seeds are indeed smaller in yeast cured by guanidine than by overexpression of Hsp104-2KT, we would expect that more monomer would be present in the yeast partially cured by guanidine than by overexpression of Hsp104-2KT. We first examined whether NGMC was in fact monomeric in lysates from [*psi^-^*] yeast by measuring its diffusion coefficient using FCS; FCS measurements on a solution of GFP were used as a control. By fitting the autocorrelation curves in Fig. 8A to a 1-component fit, dwell times were obtained for the different proteins. A dwell time of 70 µs was obtained for GFP, which yields a diffusion coefficient of 94 µm^2^ s^-1^ for GFP in solution, in agreement with other studies [36], [37]. As expected, the mobility of NGMC in [*psi^-^*] yeast lysates was slower than that of GFP in solution, with NGMC having a diffusion coefficient of 110 µs, which yields an apparent molecular weight consistent with NGMC being a monomer in solution. Apparently upon lysis of [*psi^-^*] yeast, the NGMC is no longer in a high molecular weight complex. As expected, the diffusional mobility of NGMC was an order of magnitude slower in lysates from [*PSI^+^*] yeast than from [*psi^-^*] yeast.

**Figure 9:**
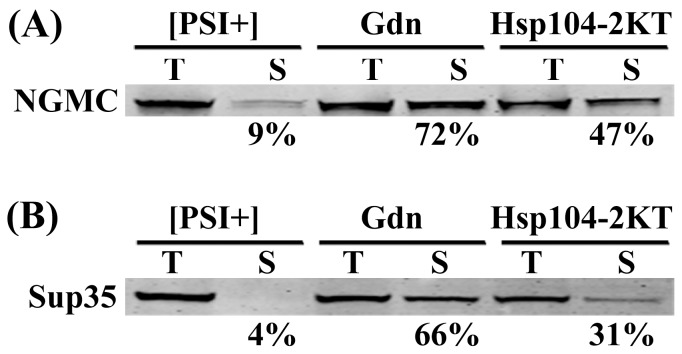
Western blot analysis of the soluble amount of NGMC and Sup35 during curing by guanidine or by overexpression of Hsp104-2KT. (A) Western blot of NGMC in total lysate (T) and supernatants (S) were from [*PSI* ^+^] strain 1074 having pFL39-GAL-HSP104KT at zero time and after growing for 5 generations either in SD with guanidine or in SGal. NGMC was probed using an anti-GFP antibody. (B) Western blot of total lysate (T) and supernatants (S) samples were from [*PSI* ^+^] strain 779-6A having pFL39-GAL-HSP104KT at zero time and after growing for 6 generations either in SD with guanidine or in SGal. Sup35 was probed using a polyclonal anti-Sup35 antibody. The percent NGMC in the supernatant indicated under each sample was corrected using Pgk1 as a loading control. Protein intensity of NGMC and Pgk1 was quantified using the Odyssey software. Western blots were performed a minimum of 3 times for each experiment and representative blots are shown in this figure.

We next examined lysates prepared from [*PSI^+^*] yeast grown for 5 generations either in 5 mM guanidine or in SGal medium to induce overexpression of Hsp104-2KT; after 5 generations of growth these yeast were still [*PSI^+^*]. Following high speed ultracentrifugation to isolate the soluble pool, Western blot analysis was used to determine the amount of soluble NGMC in the supernatants from yeast cured by the two different methods (Fig. 9A). As expected, there were only trace amounts of NGMC present in the supernatant fraction from [*PSI^+^*] yeast, whereas considerably more NGMC was present in the supernatants from partially cured yeast. Furthermore, there was significantly more NGMC in the supernatants from yeast partially cured by guanidine treatment than from yeast partially cured by overexpression of Hsp104-2KT.

Although lysates from yeast partially cured by guanidine treatment had a larger soluble pool of NGMC than lysates from yeast partially cured by overexpression of Hsp104-2KT, it was not clear whether this soluble fraction contained only monomers, small oligomers, or a mixture of the two. The aggregation state of the NGMC in the supernatants was determined using FCS to measure the diffusional mobility of the NGMC. Fig. 9B shows the autocorrelation curves of the supernatants from high speed centrifugation of lysates from yeast partially cured by guanidine treatment or by overexpression of Hsp104-2KT. After centrifugation, regardless of the method of curing, the autocorrelation curves yielded a value of 125 µs for NGMC in the supernatants. Since this value is the same as that obtained for NGMC in [*psi^-^*] cells, the NGMC in the supernatant of partially cured yeast cells is monomeric. This in turn means more monomer is present in the supernatant of lysates from cells treated with guanidine than in lysates from cells overexpressing Hsp104-2KT, which is consistent with the seeds being smaller in yeast partially cured by guanidine.

All of the above studies on curing [*PSI^+^*] by Hsp104 inactivation used GFP-labeled Sup35 (NGMC). We next examined whether native Sup35 protein shows a similar difference in the amount of soluble Sup35 depending on the method of Hsp104 inactivation. This was done by growing [*PSI^+^*] yeast strain 779-6A, the parental strain of 1074, for 6 generations in either SD medium with guanidine or in SGal medium to induce overexpression of Hsp104-2KT, at which point the plating assay showed the yeast were all still [*PSI^+^*]. Western blot analysis of yeast lysates prepared from these cells was used to determine the total amount of Sup35 in the lysates and the amount in the supernatants after high speed centrifugation. As expected based on previous observations [22], the 779-6A [*PSI^+^*] yeast strain showed less soluble Sup35, than the 1074 [*PSI^+^*] yeast strain that expresses NGMC (Fig. 9B). Nevertheless, there was more than twice as much Sup35 in the supernatant in lysates from [*PSI^+^*] yeast partially cured by guanidine than by overexpression of Hsp104-2KT (65% vs. 31%). Therefore, whether or not Sup35 is tagged with GFP, significantly more soluble Sup35 was present in [*PSI^+^*] yeast partially cured by guanidine than by overexpression of Hsp104-2KT. Since both methods show a similar time course of curing, as was shown previously by Ness et al [16], these results indicate that the reduction in seed size that occurs during curing of [*PSI^+^*] by guanidine is not dependent on the use of GFP-tagged Sup35.

## Discussion

In this study, we first established that the NGMC foci were the seeds responsible for propagating the [*PSI^+^*] prion. Specifically, regardless of whether Hsp104 was inactivated by guanidine or by overexpression of Hsp104-2KT, the fraction of [*PSI^+^*] yeast with NGMC foci directly correlated with the extent of prion curing as determined by the red/white colony assay. None of the yeast in growing culture showed large agglomerates of immobilized NGMC nor was there evidence for dead-end aggregates of NGMC; whenever cells contained visible NGMC foci, the cells and their progeny were [*PSI^+^*]. The one exception was yeast that were left with only one focus when [*PSI^+^*] was cured by overexpression Hsp104-2KT. This was not a dead-end focus because when a cell with a single focus was grown overnight in SD medium a small fraction of its daughter cells were [*PSI^+^*]. Evidently, when these cells were plated, high levels of Hsp104-2KT were still present for a time in the daughter cells resulting in the curing of most of these cells, and these cured daughter cells obscured the limited number of [*PSI^+^*] daughter cells present thus giving the colony a red appearance.

Second, we established that curing of [*PSI^+^*] by Hsp104 inactivation is due to inhibition of severing of the prion seeds by Hsp104, in agreement with the model of Tuite et al. [16], [18]. During curing by overexpression of Hsp104-2KT, the number of foci halved each generation, which shows that severing was completely inhibited and thus the seeds were diluted out by cell division. During curing by guanidine treatment, when glucose deprivation was used to make the NGMC foci apparent, our data show that severing was not completely inhibited. This accounts for the extra 2–3 generations it takes to cure [*PSI^+^*] by guanidine treatment compared to overexpression of Hsp104-2KT, as well as the difference between our data and the theoretical line obtained by halving the number of foci each generation. It is perhaps not surprising that there is residual severing activity in the presence of guanidine since in vitro experiments showed that the ATPase activity of Hsp104 was not completely inhibited by guanidine [32].

Previously, the Chernoff laboratory observed that NMG expressed from the *SUP35* promoter formed large fluorescent agglomerates during curing of [*PSI^+^*] by overexpression of Hsp104-2KT, as well as in a [*PSI^+^*] yeast strain expressing low levels of Hsp104 [7]. They proposed that the formation of large agglomerates reduced the ability of the prion to propagate, perhaps because the agglomerate cannot pass to the daughter cells. In addition, the Taguchi laboratory reported a transmission defect when guanidine was used to cure [*PSI* ^+^] in yeast expressing NMG from the *GAL1* promoter [20], which probably caused considerable overexpression of the NMG. In this latter study, the daughter cells did not inherit the large NMG foci present in the mother cells, but instead, became enriched in monomeric NMG and were cured prior to the mother cells. In contrast, when NMG was expressed from the *SUP35* promoter, which probably induced lower levels of NMG expression than the *GAL1* promoter, the Chernoff laboratory observed diffuse NMG fluorescence during curing of [*PSI^+^*] by guanidine [7], in agreement with our observations on NGMC. The fluorescent properties of NGMC in [*PSI^+^*] yeast during curing by Hsp104 inactivation was also examined by the Serio laboratory. By expressing NGMC under the *MFA1* promoter, they observed that the foci were largely immobilized when Hsp104 was inactivated either by guanidine treatment or by overexpression of Hsp104-2KT [19]. The differences between these various studies may be due to experimental conditions including the time of curing, the amount of expression, and the construct that was GFP-labeled.

Studies overexpressing NMG suggest it forms large agglomerates that cannot be significantly reduced in size or transmitted to daughter cells. With regards to this study, we examined whether there was a defect in the transmission of the NGMC foci to daughter cells during curing of [*PSI^+^*] by overexpression of Hsp104-2KT. Our data clearly show that the foci are transmitted based on their abundance in the daughter cells relative to the volume of the mother and daughter cells, as proposed by the Byrne et. al. model [18]. On the other hand, even in yeast that are propagating the [*PSI^+^*] prion, Serio’s laboratory has detected a defect in the transmission of seeds to daughter cells [31]. Several factors might account for the fact that we do not detect a size bias in transmission of seeds during the curing that occurs when Hsp104 activity is reduced. First, in untreated [*PSI^+^*] yeast, Hsp104 is actively remodeling the seeds, which could make the seed size heterogeneous, whereas when Hsp104-2KT is overexpressed, there is no remodeling of the seeds and all the seeds tend to be large [19]. Just based on diffusion alone, if smaller foci were present, they would diffuse faster and be more likely to pass from mother to daughter than larger foci. In addition, the polarisome might play a role in causing the large foci to be retained in the mother cell. The polarisome is involved in an Hsp104-dependent mechanism for transporting misfolded proteins from the daughter to the mother along actin cables [39]. If the polarisome plays an important role in yeast with active Hsp104 as opposed to yeast in which Hsp104 activity is inhibited, it could cause retention of large foci in the mother cells.

Although the mechanism of curing by inactivation of Hsp104 by guanidine treatment and by overexpression of Hsp104-2KT was similar, our data show that the NGMC foci became undetectable during curing by guanidine treatment. The fact that the NGMC appeared diffusive during curing by guanidine even though the cells were still [*PSI^+^*], led us to incorrectly propose that cell division was not essential for the curing of [*PSI^+^*] [21]. However, even though the seeds were not visible, we have now shown that the NGMC foci became readily apparent when the [*PSI^+^*] yeast were either starved or Hsp104-2KT was overexpressed. Since severing activity is 80% inhibited in 5 mM guanidine, severing activity cannot account for all of the seeds becoming undetectable. Furthermore, when [*PSI^+^*] was cured by overexpression of Hsp104(K218T) in the presence of guanidine, the curing kinetics showed severing was completely inhibited, but still the seeds became undetectable. This again shows that severing is not causing the absence of detectable foci.

FCS microscopy observations suggested that the loss of detectable foci in [*PSI^+^*] yeast during curing by guanidine treatment is due to a reduction in the size of the foci. This reduction in size is dependent on Hsp104 since yeast in which the *HSP104* gene was conditionally deleted showed prominent NGMC foci during curing of [*PSI^+^*] both in the absence and presence of guanidine. Therefore, our data are consistent with Hsp104 having an activity in the presence of guanidine that reduces or “trims” the size of the prion seeds without severing them into new seeds. This trimming activity was also observed when [*PSI^+^*] was cured by overexpression of Hsp104(K218T), which contains only a point mutation at the ATPase site of the NBD1 domain of Hsp104. Apparently, trimming activity removes Sup35 molecules from the prion seeds, but these released Sup35 molecules do not form a new seed. In fact, a size threshold for misfolded Sup35 oligomers to propagate new seeds rather than to dissolve into monomer has been incorporated into previous models of prion propagation [31], [40]. One possibility is that during trimming, Hsp104 extracts Sup35 molecules from the ends of the prion fiber, whereas during severing, it extracts Sup35 molecules from the interior of the prion fiber. Therefore, during trimming from the ends of the amyloid fiber, the released Sup35 oligomers may dissolve into monomer, whereas during severing a new seed is formed.

Not only does trimming by Hsp104 occur during curing of [*PSI^+^*] by guanidine, but there is also evidence that trimming occurs in propagating yeast. When Ssa1 was overexpressed, the time course of [*PSI^+^*] curing by guanidine treatment was not affected, but the NGMC foci remained prominent until the yeast were cured. This shows that SSa1 does not affect severing in the presence of guanidine even though it markedly reduces trimming. If trimming also occurs in propagating [*PSI^+^*] yeast, Ssa1 should have the same effect here, that is, without affecting severing it should increase the size of the foci. Indeed this is what occurred in the 1074 yeast strain of propagating [*PSI^+^*] yeast. Ssa1 markedly increased the size of the foci without curing [*PSI^+^*] even after 20 generations of Ssa1 overexpression. These results are consistent with inhibition of trimming of prion seeds in [*PSI^+^*] yeast in the presence of excess Ssa1. As for why the foci are larger in [*PSI^+^*] yeast with active Hsp104 than in [*PSI^+^*] yeast partially cured by guanidine, this may occur because as Hsp104 severs the prion seeds, there may be less Hsp104 available to trim the seeds.

Our proposal that Hsp104 both severs and trims seeds is in agreement with observations that have been made on other triple-A ATPases. In mammalian cells, two different triple-A ATPase proteins, spastin and katinin, are able to both trim and sever microtubules [41]. However, as part of their trimming activity these proteins are able to completely depolymerize microtubules, and if trimming alone were able to completely depolymerize prion seeds, incubation of [*PSI^+^*] cells in the presence of guanidine would be expected to cure yeast even if cell division were prevented. Of course, as we pointed out above, this did not occur in our previous experiments, suggesting that perhaps the prion core has a special structure that makes it resistant to trimming. Finally, not only inactivation of Hsp104 cures [*PSI^+^*] prion, but overexpression of Hsp104 also cures [2]. Since it has been previously been observed that during the curing of [*PSI^+^*] by overexpression of Hsp104, Hsp104 partially solubilizes Sup35 aggregates [42], it may turn out that the trimming activity of Hsp104 is important for curing by overexpression of Hsp104.

## Supporting Information

Movie S1 **Transmission of the prion aggregates NGMC foci from mother to daughter cell during curing by Hsp104-2KT overexpression.** Cells of 1074 strain having pFL39-GAL-HSP104KT were grown for 6–7 generations in SGal and imaged using Zeiss LSM780 confocal microscope. After first imaging the daughter cell, it was photobleached for 5 s, and then imaged every second for the next 2 min. More than 20 budding cells were imaged and a representative one is shown in this movie.
(AVI)

## References

[1] Wickner RB, Edskes HK, Maddelein ML, Taylor KL, Moriyama H (1999). Prions of yeast and fungi. Proteins as genetic material.. J Biol Chem.

[2] Chernoff YO, Lindquist SL, Ono B, Inge-Vechtomov SG, Liebman SW (1995). Role of the chaperone protein Hsp104 in propagation of the yeast prion-like factor [*PSI+*].. Science.

[3] Moriyama H, Edskes HK, Wickner RB (2000). [URE3] Prion Propagation in Saccharomyces cerevisiae: Requirement for Chaperone Hsp104 and Curing by Overexpressed Chaperone Ydj1p.. Mol Cell Biol.

[4] Lee S, Sowa ME, Choi JM, Tsai FT (2004). The ClpB/Hsp104 molecular chaperone–a protein disaggregating machine.. J Struct Biol.

[5] Tuite MF, Mundy CR, Cox BS (1981). Agents that cause a high frequency of genetic change from [*PSI+*] to [*psi-*] in *Sacchromyces cerevisiae*.. Genetics.

[6] Jung G, Masison DC (2001). Guanidine hydrochloride inhibits Hsp104 activity in vivo: a possible explanation for its effect in curing yeast prions.. Curr Microbiol.

[7] Wegrzyn RD, Bapat K, Newnam GP, Zink AD, Chernoff YO (2001). Mechanism of prion loss after Hsp104 inactivation in yeast.. Mol Cell Biol.

[8] Patino MM, Liu JJ, Glover JR, Lindquist S (1996). Support for the prion hypothesis for inheritance of a phenotypic trait in yeast.. Science.

[9] Borchsenius AS, Wegrzyn RD, Newnam GP, Inge-Vechtomov SG, Chernoff YO (2001). Yeast prion protein derivative defective in aggregate shearing and production of new 'seeds'.. EMBO J.

[10] DePace AH, Santoso A, Hillner P, Weissman JS (1998). A critical role for amino-terminal glutamine/asparagine repeats in the formation and propagation of a yeast prion.. Cell.

[11] Zhou P, Derkatch IL, Liebman SW (2001). The relationship between visible intracellular aggregates that appear after overexpression of Sup35 and the yeast prion-like elements [*PSI+*] and [*PIN+*].. Mol Microbiol.

[12] Song Y, Wu YX, Jung G, Tutar Y, Eisenberg E (2005). Role for Hsp70 chaperone in Saccharomyces cerevisiae prion seed replication.. Eukaryot Cell.

[13] Satpute-Krishnan P, Serio TR (2005). Prion protein remodelling confers an immediate phenotypic switch.. Nature.

[14] Chernoff YO (2004). Cellular control of prion formation and propagation in yeast. Telling GC, editor.. Prions and Prion Diseases: Current Perspectives Norfolk, England: Horizon Biosciences.

[15] Ferreira PC, Ness F, Edwards SR, Cox BS, Tuite MF (2001). The elimination of the yeast [PSI+] prion by guanidine hydrochloride is the result of Hsp104 inactivation.. Mol Microbiol.

[16] Ness F, Ferreira P, Cox BS, Tuite MF (2002). Guanidine hydrochloride inhibits the generation of prion “seeds” but not prion protein aggregation in yeast.. Mol Cell Biol.

[17] Eaglestone SS, Ruddock LW, Cox BS, Tuite MF (2000). Guanidine hydrochloride blocks a critical step in the propagation of the prion-like determinant [*PSI+*] of *Saccharomyces cerevisiae*.. Proc Natl Acad Sci USA.

[18] Byrne LJ, Cox BS, Cole DJ, Ridout MS, Morgan BJ (2007). Cell division is essential for elimination of the yeast [*PSI+*] prion by guanidine hydrochloride.. Proc Natl Acad Sci U S A.

[19] Satpute-Krishnan P, Langseth SX, Serio TR (2007). Hsp104-dependent remodeling of prion complexes mediates protein-only inheritance.. PLoS Biol.

[20] Kawai-Noma S, Pack C-G, Tsuji T, Kinjo M, Taguchi H (2009). Single mother–daughter pair analysis to clarify the diffusion properties of yeast prion Sup35 in guanidine-HCl-treated [*PSI+*] cells.. Genes Cells.

[21] Wu YX, Greene LE, Masison DC, Eisenberg E (2005). Curing of yeast [*PSI+*] prion by guanidine inactivation of Hsp104 does not require cell division.. Proc Natl Acad Sci U S A.

[22] Mathur V, Hong JY, Liebman SW (2009). Ssa1 Overexpression and [*PIN+*] variants cure [*PSI+*] by dilution of aggregates.. J Mol Biol.

[23] Gietz RD, Schiestl RH (2007). Large-scale high-efficiency yeast transformation using the LiAc/SS carrier DNA/PEG method.. Nat Protocols.

[24] Park YN, Masison D, Eisenberg E, Greene LE (2011). Application of the FLP/FRT system for conditional gene deletion in yeast Saccharomyces cerevisiae.. Yeast.

[25] Hung G-C, Masison DC (2006). N-terminal domain of yeast Hsp104 chaperone is dispensable for thermotolerance and prion propagation but necessary for curing prions by Hsp104 overexpression.. Genetics.

[26] Jung G, Jones G, Wegrzyn RD, Masison DC (2000). A role for cytosolic hsp70 in yeast [[*PSI+*] prion propagation and [*PSI+*] as a cellular stress.. Genetics.

[27] Greene LE, Park YN, Masison DC, Eisenberg E (2009). Application of GFP-labeling to study prions in yeast.. Protein Pept Lett.

[28] Cox B, Ness F, Tuite M (2003). Analysis of the generation and segregation of propagons: entities that propagate the [*PSI+*] prion in yeast.. Genetics.

[29] Ghaemmaghami S, Huh W-K, Bower K, Howson RW, Belle A (2003). Global analysis of protein expression in yeast.. Nature.

[30] Byrne LJ, Cole DJ, Cox BS, Ridout MS, Morgan BJT (2009). The number and transmission of [*PSI+*] prion seeds (propagons) in the yeast *Saccharomyces cerevisiae*.. PLoS ONE.

[31] Derdowski A, Sindi SS, Klaips CL, DiSalvo S, Serio TR (2010). A size threshold limits prion transmission and establishes phenotypic diversity.. Science.

[32] Grimminger V, Richter K, Imhof A, Buchner J, Walter S (2004). The prion curing agent guanidinium chloride specifically inhibits ATP hydrolysis by Hsp104.. J Biol Chem.

[33] Schirmer EC, Ware DM, Queitsch C, Kowal AS, Lindquist SL (2001). Subunit interactions influence the biochemical and biological properties of Hsp104.. Proc Natl Acad Sci U S A.

[34] Newnam GP, Wegrzyn RD, Lindquist SL, Chernoff YO (1999). Antagonistic Interactions between Yeast Chaperones Hsp104 and Hsp70 in Prion Curing.. Mol Cell Biol.

[35] Kawai-Noma S, Ayano S, Pack C-G, Kinjo M, Yoshida M (2006). Dynamics of yeast prion aggregates in single living cells.. Genes Cells.

[36] Elsner M, Hashimoto H, Simpson JC, Cassel D, Nilsson T (2003). Spatiotemporal dynamics of the COPI vesicle machinery.. EMBO Rep.

[37] Wang Z, Shah JV, Chen Z, Sun CH, Berns MW (2004). Fluorescence correlation spectroscopy investigation of a GFP mutant-enhanced cyan fluorescent protein and its tubulin fusion in living cells with two-photon excitation.. J Biomed Opt.

[38] Bagriantsev SN, Gracheva EO, Richmond JE, Liebman SW (2008). Variant-specific [*PSI+*] infection is transmitted by Sup35 polymers within [*PSI+*] aggregates with heterogeneous protein composition.. Mol Biol Cell.

[39] Erjavec N, Larsson L, Grantham J, Nystrom T (2007). Accelerated aging and failure to segregate damaged proteins in Sir2 mutants can be suppressed by overproducing the protein aggregation-remodeling factor Hsp104p.. Genes Dev.

[40] Tanaka M, Collins SR, Toyama BH, Weissman JS (2006). The physical basis of how prion conformations determine strain phenotypes.. Nature.

[41] Roll-Mecak A, Vale RD (2008). Structural basis of microtubule severing by the hereditary spastic paraplegia protein spastin.. Nature.

[42] Paushkin SV, Kushnirov VV, Smirnov VN, Ter-Avanesyan MD (1996). Propagation of the yeast prion-like [*PSI+*] determinant is mediated by oligomerization of the SUP35-encoded polypeptide chain release factor.. EMBO J.

